# Relationship Between Pituitary Siderosis and Endocrinological Disorders in Pediatric Patients with Beta-Thalassemia

**DOI:** 10.7759/cureus.12877

**Published:** 2021-01-23

**Authors:** Kamil Yılmaz, Ahmet Kan, Mehmet Guli Çetincakmak, V.Hulya Uzel, Deniz Yılmaz, Muhammed Akif Deniz, Salih Hattapoglu

**Affiliations:** 1 Pediatrics, Dicle University School of Medicine, Diyarbakir, TUR; 2 Pediatric Allergy, Dicle University School of Medicine, Diyarbakir, TUR; 3 Radiology, Memorial Regional Hospital, Diyarbakir, TUR; 4 Pediatric Oncology, Dicle University School of Medicine, Diyarbakir, TUR; 5 Pathology, Dicle University School of Medicine, Diyarbakir, TUR; 6 Radiology, Dicle University School of Medicine, Diyarbakir, TUR

**Keywords:** beta thalassemia, pituitary mri, t2, siderosis

## Abstract

Introduction

Excess iron accumulation occurs mainly in organs such as reticuloendothelial cells, the pituitary gland, and the pancreas in beta-thalassemia because of blood transfusions. In the present study, it was aimed to investigate the relationship between T2* values on magnetic resonance imaging (MRI) and clinically diagnosed pituitary endocrinological disorders in children with thalassemia major.

Methods

This study enrolled patients diagnosed with beta-thalassemia at pediatric hematology outpatient clinics. In the study, in addition to the medical history of the patients, routinely performed tests, including hemoglobin electrophoresis, routine biochemical tests, and tests for pubertal development (follicle-stimulating hormone (FSH), luteinizing hormone (LH), estradiol, testosterone, etc.), as well as iron deposition measured by hepatic MRI T2* (STAR) sequence, were retrospectively assessed. A total of 29 patients were enrolled.

Results

Hypothyroidism was detected in 34.6% (9/26) of patients, short stature in 37% (10/27), and pubertal retardation in 50% (14/28) of the patients. There was no significant correlation between hypothyroidism and pituitary MRI T2* values. No significant correlation was found between laboratory parameters and pituitary MRI examination. Although the sensitivity of T2* levels could rise above 80%, their specificity remained low. This is one of the major limitations of the pituitary MR T2* study for the prediction of short stature. The best lower cut-off level of MR T2* to predict short stature was found 14.6 ms.

Conclusion

The diagnostic specificity pituitary MR examination levels for short stature were detected as low. Thus, the clinical standardization and validation of pituitary MR T2* values examination are needed before clinical follow-up and multifaceted studies are needed.

## Introduction

Beta-thalassemia is a hereditary disease characterized by ineffective erythropoiesis as a result of a defect in the synthesis of mature erythrocytes [1]. The standard therapy for beta-thalassemia is blood transfusion. As a result of blood transfusions, excess iron accumulation occurs mainly in organs such as reticuloendothelial cells, the pituitary gland, and the pancreas. Iron deposition may cause serious and sometimes fatal complications [2]. Pituitary dysfunction and hypogonadotropic hypogonadism due to iron deposition are the most common endocrinological complications despite adequate chelation therapy [3]. Adequate function of the anterior pituitary gland is critical for growth and sexual development during the adolescence period, and it affects directly the quality of life and growth of young children with thalassemia major [3]. In patients with thalassemia, pubertal delay, sexual dysfunction, and/or infertility may occur at rates ranging between 51% and 80% as a result of transfusions [4-5]. It is important to detect iron deposition in the pituitary gland because hypogonadism may be reversed by intense chelation therapy [6]. Hypogonadism may result from damaged gonads (primary), the pituitary gland (secondary), or the hypothalamus (tertiary). Secondary hypogonadism (also called hypogonadal hypogonadism) is the most common cause of hypogonadism in thalassemia major [6-7]. It is difficult to detect pituitary insufficiency clinically before puberty because of the immaturity of the hypothalamic-pituitary-gonadal axis [8]. Therefore, studies detecting pituitary iron deposition before symptoms may be valuable for the early diagnosis of patients who are at risk.

In patients with thalassemia major, a reduction of the target height, growth retardation, and pubertal delay/arrest are also common. Short stature is mostly due to a short body length, and it starts clinically at five to six years of age in boys and after eight years of age in girls. Progressive loss in linear growth usually continues until puberty when physiological growth attack is usually decreased and/or delayed. Then, growth retardation becomes more prominent [9-11].

The etiopathogenesis of growth retardation is not entirely clear. Chronic anemia due to chronic tissue hypoxia, siderosis, bone dysplasia due to chelation toxicity [12-14], zinc deficiency, hypothyroidism, hypogonadism/delayed puberty, and dysfunctional GHRH-IGF-1 growth axis have been implicated [8,15]. In addition, the transfusion regimen and the mode of iron chelation therapy interact with these factors [8]. Patients may often start to be affected at a young age and irreversible endocrinological deficiency may be prevented by early and rapid diagnosis [7].

It is long known that the evaluation of T2* signal evaluation in magnetic resonance (MR) imaging is an important diagnostic modality for the detection of iron deposition in organs. Iron deposition in tissues causes a significant reduction in T2* relaxation time and signal intensity [16-17]. Iron accumulation is detected by measuring the signal loss detected on images by different techniques in MR [17]. T2-star (T2*) is currently the most commonly employed non-invasive method to detect iron deposition in the cardiac and hepatic tissues.

Magnetic resonance (MR)-based diagnostic approaches have been standardized and validated for cardiac, hepatic, and pancreatic iron deposition [18-19]. In the literature, MR imaging studies on iron deposition in the pituitary gland are not sufficient for standardization and validation [19-20]. In patients with beta-thalassemia major, serum follicle-stimulating hormone (FSH), luteinizing hormone (LH), testosterone, thyroid-stimulating hormone (TSH), and prolactin measurements may be important for diagnosing delayed or arrested puberty or hypogonadism. A low testosterone level is one of the best parameters that reflects the pituitary-pubertal developmental axis in the pubertal period. An increase in FSH and/or LH is helpful for differentiating primary testicular failure from the secondary form. Free T3 (fT3), free T4 (fT3), and TSH should be checked to screen thyroid function [21].

In patients with thalassemia, pituitary insufficiency, such as gonadal failure, hypothyroidism, and growth retardation, which develop as a result of iron deposition may be detected by a reduction of MR T2* signals at an early period. In the present study, it was aimed to investigate the relationship between a reduced signal intensity on T2* weighted images on MR and clinically diagnosed pituitary endocrinological disorders in children with thalassemia major.

## Materials and methods

This study enrolled patients of both genders who were diagnosed with beta-thalassemia at pediatric hematology outpatient clinics between January 2010 and June 2020 who received regular transfusion and iron chelation therapy. The patients were routinely taking their chelation therapies, alone or in combination. In the study, in addition to the medical history of the patients, routinely performed tests, including hemoglobin electrophoresis, routine biochemical tests, and tests for pubertal development (FSH, LH, estradiol, testosterone, etc.), as well as iron deposition measured by hepatic, cardiac and pituitary MR T2* (STAR) sequence, were retrospectively assessed. A total of 29 patients were enrolled. Patients’ medical history (blood transfusion regimen and chelation therapy), growth percentile, and pubertal stage (by evaluating weight, height, body mass index, and Tanner stage) were separately evaluated. The investigated laboratory parameters included the measurements of the serum levels of ferritin, hemoglobin, TSH, free T4 (fT4), free T3 (fT3), FSH, LH, testosterone, and estradiol.

The diagnosis of hypothyroidism was made by evaluating the TSH, fT4, and fT3 values according to age.

Diagnosis of delayed puberty and hypogonadism

Delayed puberty was defined as the absence of pubertal development by the age of 13 years in girls and 14 years in boys. Hypogonadism was defined as the absence of testicular growth (smaller than 4 millimeters) in boys and the absence of breast development by the age of 16 years in girls.

Diagnosis of short stature

Short stature was defined as a mean height below 2 S.D. by age and sex [22].

Exclusion criteria

Exclusion criteria included children whose growth or pubertal development could not be accessed in the screening of medical records; children taking hormone therapy for growth retardation; children who were <13 years old for girls and <14 years old for boys; children with other chronic systemic disorders that may cause short stature (severe hepatic, renal, or cardiac disorders, diabetes mellitus, severe infections).

MR T2* assessments of the liver, heart, and pituitary, as well as endocrinological evaluation of the subjects, were performed cross-sectionally at the time (or described time interval) of MRI scanning.

Statistical method

Statistical analyses were performed using the Social Package for the Social Sciences (SPSS) 22 software package (IBM Corp., Armonk, NY). The normality of the distribution of the study variables was tested by visual (histograms and probability graphics) and analytic methods. Descriptive statistics included median for non-normally distributed variables, mean for normally distributed variables, and frequency for ordinal variables. Spearman’s test was used to determine the correlation coefficients and statistical significance of the correlations between variables, at least one of which was non-normally distributed or was an ordinal variable. The diagnostic predictive characteristics of MR T2* value for the presence of short stature were analyzed using Receiver Operating Characteristics (ROC) curve analysis.

## Results

The sociodemographic properties of the patients included in the study are shown in Table 1; 58.6% were female. Pituitary, cardiac, and liver MR T2* values and the laboratory results of the patients are shown in Table 2 and Table 3.

**Table 1:** Sociodemographic characteristics of the patients

Parameters	Median	Min-max	n=29
Age (year)	15	13-18	29
Weight (kg)	46.1	28.7-52	29
Height (cm)	144	131-158.5	29

**Table 2:** Evaluation of patients MRI results S.D.: standard deviation, MRI: magnetic resonance imaging

Examination	T2 values (ms), (mean±S.D.)
Pituitary MRI (ms)	12.8±4.34
Liver MRI (ms)	3.46±0.55
Cardiac MRI (ms)	33.3±7.1

**Table 3:** Hormonal evaluation of patients *Reference ranges vary by age, TSH: thyroid-stimulating hormone, FSH: follicle-stimulating hormone, LH: luteinizing hormone, n: number of patients, fT4: free T4, fT3: free T3 ref.: references

Parameters	mean±s.d	n
Serume ferritin (ng/ml), ref.:(10-291)	1984.5±714.5	29
TSH (µIU/mL), ref.:(0.35-5.5)	2.99±1.19	26
fT4 (ng/mL), ref.: 0.89-1.76	1.64±0.51	26
fT3 (pmol/L), ref.: (2.3-4.2)	3.9±1.5	26
*FSH (mIU/ml)	2.4±1.7	27
*LH (mIU/ml)	3.3±2.9	27
*Testosterone (ng/mL)	0.1±0.08	16
*Estradiol (pg/mL)	57.5±88.6	12

Hypothyroidism was detected in 34.6% (9/26) of patients, short stature in 37% (10/27), and pubertal retardation in 50% (14/28). The most commonly encountered endocrinological disorder was pubertal retardation. The frequencies of the endocrinological disorders of the patients are presented in Table 4.

**Table 4:** Frequency of endocrine disorders detected in patients *number of patients examined

Endocrine disease	%
Hypothyroidism, (n=9/26*)	34.6
Short stature, (n=10/27*)	37
Puberty retardation (n=14/28*)	50

**Figure 1:**
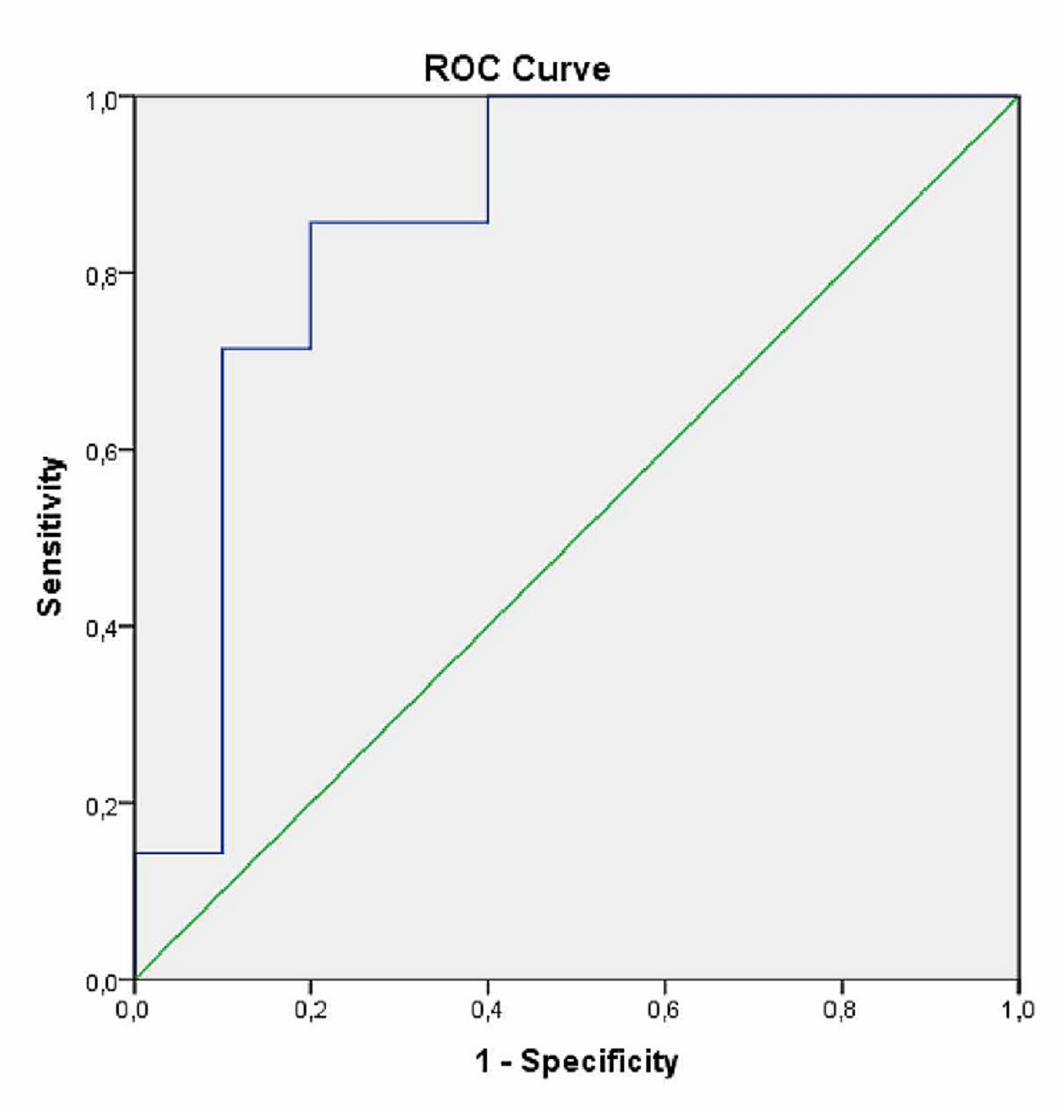
ROC curve of the MRI T2 signal used to predict the presence of short stature MRI: magnetic resonance imaging, ROC: receiver operating characteristics

The diagnostic predictive characteristics of the MR T2* value for the presence of short stature were examined by an ROC curve (Figure 1). In the presence of significant threshold values, the sensitivity and specificity figures of those thresholds were calculated (Table 5). In the resulting graphics, it was found that the pituitary MR T2* value was significantly higher than the reference line, with the value having a significant diagnostic value (p=0.015). It was found that even when the sensitivity of the MR T2* value for detecting short stature reached about 80%, its specificity remained low. The cut-off values obtained by examining the curve are shown in Table 5.

**Table 5:** Cutoff values for pituitary MRI T2 values in detecting short stature MRI: magnetic resonance imaging

Limit Value for MRI T2 Signal	Sensitivity (%)	Specificity (%)
14.6 ms	87.5 %	40 %
14.3 ms	87.5 %	30 %

There was no significant correlation between the pituitary T2* signal and hepatic and cardiac T2* values. No significant correlation was found between laboratory parameters and pituitary MR examination (Table 6).

**Table 6:** Correlation analysis between pituitary MRI T2 and parameters TSH: thyroid-stimulating hormone, FSH: follicle-stimulating hormone, LH: luteinizing hormone, n: number of patients, fT4: free T4, fT3: free T3 ref.: references, MRI: magnetic resonance imaging

Parameters	Correlation Coefficient	p	n
Ferritin (n:17)	-0.91	0.72	17
TSH (n:17)	-0.35	0.17	17
fT4 (n:17)	0.39	0.13	17
fT3 (n:17)	-0.15	0.55	17
FSH (n:17)	-0.026	0.92	17
LH (n:17)	0.21	0.51	17
Testosteron (n:16)	0.27	0.38	16
Estradiol (n:12)	0.23	0.61	12
Age (n:29)	0.25	0.71	17
Heart MRI T2 values (n:12)	0.17	0.58	12
Liver MRI T2 values (n:12)	0.12	0.69	12

## Discussion

Iron deposition is common in thalassemia major due to treatments such as frequent blood transfusions, appropriate chelation therapy, and bone marrow transplantation. Various complications, including severe endocrinopathies, cardiomyopathies, and bone disorders, are commonly seen in these patients [23].

Noetzli et al. studied 47 patients with thalassemia major and five patients with other blood disorders, with a mean age of 20.4±12.1 years, who received blood transfusions. They reported that iron deposition and volume loss increased with advanced age. They also detected that iron deposition that starts early in life markedly accelerates in the second half of life [24].

De Sanctis et al. studied 3817 patients with thalassemia major, of whom 38% were 16 years of age or older [22]. The most common endocrinological complication that the authors found was pubertal delay. They found that growth hormone deficiency occurred at a rate of 7.9% in males and 8.8% in females while hypothyroidism had a prevalence of 3.2%. On the other hand, in a cross-sectional study by Saffari et al., which included 77 patients with thalassemia major who were 15-36 years old, the most common endocrinological disorders were pubertal delay/arrest (46.8%), followed by short stature (33.8%) and (15.9%) hypothyroidism [25]. Shamshirsaz et al. reported that short stature was the most common endocrinological disorder (39.3%) among 220 patients with thalassemia major and a mean age of 15.2 ± 3.1 years. They found hypogonadism in 22.9% of boys and 12.2% of girls while they found primary hypothyroidism in 7.7% of total patients [21].

As is seen, the prevalences of endocrinological disorders seen in thalassemia major vary considerably between studies. Differences in treatment protocols and age ranges of the study groups may be affecting the study results. The most common endocrinological complications in our study were pubertal delay (50%) followed by short stature (37%) and hypothyroidism (34.6%).

Mousa et al. compared 38 patients with thalassemia who were older than 17 years with a control group. They found that as pituitary T2* signal intensity was reduced, it was more likely to be correlated with hypogonadotropic gonadism and short stature [26]. In addition, they showed that T2* values were correlated to serum ferritin levels. In a study by Lau et al. that was conducted on 12 children with thalassemia aged three to 21 years, no correlation was found between serum ferritin level and pituitary MR T2* levels [27]. Çetinçakmak et al. studied 80 patients aged four to 34 years who had thalassemia major. They found that hepatic, cardiac, and pituitary MR T2* levels were negatively correlated to serum ferritin level and age. In our study, no correlation was found between pituitary MR T2* levels and serum ferritin level. Considering the literature data and our study results, the relationship between ferritin and pituitary MR examination is unclear [28]. There is a need for large population studies to elucidate this subject in detail. It has been shown by various studies that pituitary iron deposition is correlated with hepatic MR and cardiac MR T2* levels. In a study on adults (≥17 years) with thalassemia, it was shown that there was a correlation between hepatic iron deposition and pituitary iron deposition [26]. In our study, there wasn’t any significant correlation between pituitary MR T2* signal intensity levels and hepatic or cardiac MR T2* levels. The absence of a correlation can be explained by the difference in iron’s pharmacokinetic properties. The pituitary gland, like the heart, takes circulating iron not bound to transferrin while iron metabolism in the liver is largely regulated by transferrin. In addition, not all patients in our study group could undergo hepatic and cardiac MR examination, and our study population was small. So, this correlation could not be examined homogeneously in our study.

It is recommended that the initial hormone tests should include FSH, LH, testosterone, and TSH in order to detect delayed puberty or hypogonadism in patients with thalassemia [3,22,29]. Therefore, these tests were ordered as the baseline tests in our patients. No statistically significant correlation was shown between the laboratory tests and pituitary MR T2* values (Table 6).

There are many studies in the literature on T2* level changes due to pituitary iron deposition. Çetinçakmak et al. found a mean MR T2* level below 14.9 ms among 84 thalassemia patients with an age range of four to 34 years [28]. In another study, the mean MR T2* level was less than 5.9 ms in 180 patients with thalassemia major whose ages ranged between 12 and 48 years [30]. Our study found a mean MR T2* level of 12.8±4.34 ms. The discrepancy between the results of the available studies can be explained by the differences between the population size and age range of the participants. The diagnostic predictive power of pituitary MR examination levels for short stature was determined by the analysis of the ROC curve. Although the sensitivity of T2* levels could rise above 80%, their specificity remained low. This is one of the major limitations of a pituitary MR T2* study for the prediction of short stature. The best lower cut-off level of MR T2* to predict short stature was found to be 14.6 ms (Table 5).

One of the limitations of our study was its small sample size. In addition, it is difficult to make any implications for the effect of chelators on pituitary iron deposition in a retrospective study. This effect could vary by personal factors, age, a patient’s clinical status, and the chelation therapy used. It is difficult to ascertain the total number of transfusions in our patients. We believe that the figure may have affected the results.

## Conclusions

Based on our study, no statistically significant relationship was obtained between laboratory tests and pituitary MR T2* values. The diagnostic specificity pituitary MR examination levels for short stature were detected as low. Thus, clinical standardization and the validation of pituitary MR T2* values examination are needed before clinical follow-up and multifaceted studies are also needed.
